# A Multi‐Tissue Transcriptomic Resource for the Brahminy Blindsnake *Indotyphlops braminus* (Squamata: Typhlopidae)

**DOI:** 10.1002/ece3.74602

**Published:** 2026-10-28

**Authors:** Kexin Sun, Xiang Ji, Jing Lu, Anqiong Lu, Yuqian Wang, Fei Zhu

**Affiliations:** ^1^ School of Life Sciences Guizhou Normal University Guiyang Guizhou China

**Keywords:** *Indotyphlops braminus*, parthenogenesis, RNA‐seq, transcriptome

## Abstract

The Brahminy blindsnake ( *Indotyphlops braminus* ), an all‐female, parthenogenetic, and triploid snake, is an informative species for studying polyploidy and asexual reproduction in vertebrates. In this study, we analyzed tissue expression patterns in *I. braminus* using a PacBio‐derived transcript reference and Illumina RNA‐seq. Differential expression and annotation over‐representation analyses were used to characterize tissue‐associated expression differences and over‐represented annotations across ovary, heart, liver, and skin from the same three individuals. The three overlapping eye‐spot pools were described at the library level. Weighted gene co‐expression network analysis (WGCNA) was used only as an exploratory analysis of the 12 four‐tissue libraries. A total of 835,353,682 clean read pairs were retained, and gene expression profiles showed broad differences among the four formally analyzed tissues. Of 30,224 tested genes, 13,849 showed a tissue effect, and 4228 candidate tissue‐enriched genes were identified by requiring higher expression in all three relevant pairwise comparisons. Six representative annotation terms remained significant in the sensitivity analysis, and 3 of 11 non‐gray co‐expression modules passed the prespecified stability rule across nine alternative networks. This study provides an additional multi‐tissue transcriptomic resource for *I. braminus* , with candidate sets that can guide future functional and comparative tests of hypotheses concerning parthenogenesis, triploidy, fossoriality, and visual reduction.

## Graphical abstract

We analyzed multi‐tissue expression patterns in the all‐female, parthenogenetic, and triploid Brahminy blindsnake ( *Indotyphlops braminus* ) using PacBio and Illumina RNA‐seq to better understand polyploidy and asexual reproduction in vertebrates. By characterizing differential expression and co‐expression networks across the ovary, heart, liver, skin, and eye‐spot tissues, we identified thousands of tissue‐enriched genes and stable gene modules. This study provides a valuable multi‐tissue transcriptomic resource to guide future functional and comparative studies on parthenogenesis, triploidy, fossoriality, and visual reduction.

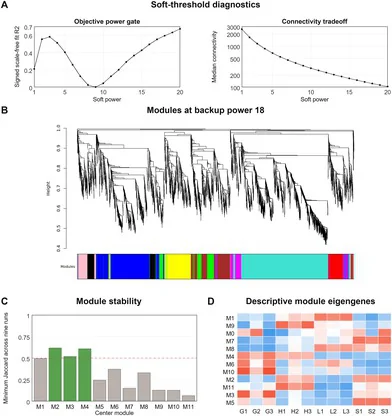

## 1 Introduction

The Brahminy blindsnake, *Indotyphlops braminus* (Typhlopidae), is a small fossorial snake with a slender, cylindrical body, and reduced eyes (Wallach 2009). It is an all‐female, parthenogenetic snake (Figure 1). McDowell first proposed that the species was all female, and later accounts explicitly reported that this inference was based on 114 specimens examined without finding a male (Mcdowell 1974; Ota et al. 1991; Wallach 2009). A few early reports mentioned putative males (Wall 1918, 1921; List 1958), although a later account suggested that these records probably concerned similar‐looking typhlopids (Wallach 2009). Later cytogenetic studies reported 42 chromosomes arranged in 14 triplets, supporting a triploid interpretation of this unisexual lineage (Wynn et al. 1987; Ota et al. 1991). Specimens from the Ryukyu Archipelago and Taiwan were karyotyped, but the cellular or genetic basis of parthenogenesis and triploid maintenance remained unresolved (Ota et al. 1991). Parthenogenetic vertebrates do not all use the same cytological route, and even detailed meiotic findings from other reptiles cannot simply be transferred to *I. braminus* (Kearney et al. 2009; Lutes et al. 2010; Neaves and Baumann 2011; Booth and Schuett 2016; Newton et al. 2016; Fujita et al. 2020; Dedukh et al. 2022, 2024).

**FIGURE 1:**
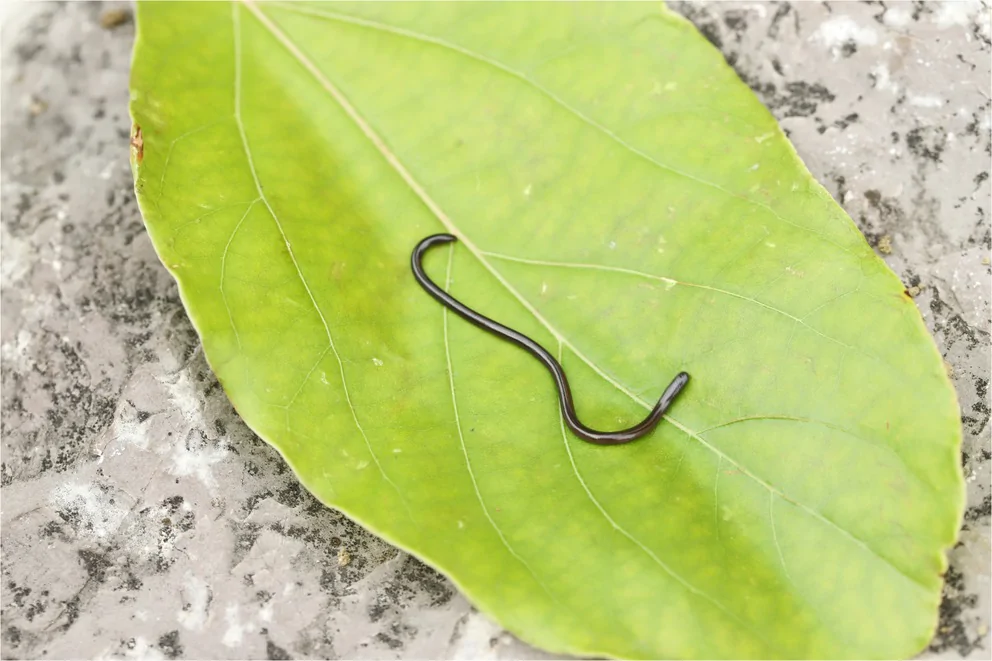
A Brahminy blindsnake ( *Indotyphlops braminus* ) collected from Wangmo County, Guizhou Province, China.

Genome resources now cover several snake lineages (Castoe et al. 2011; Duan et al. 2017; Suryamohan et al. 2020; Kolora et al. 2021). Transcriptome studies have also compared expression among tissues in snakes and other reptiles (Schwartz et al. 2010; Eckalbar et al. 2013; Reyes‐Velasco et al. 2015; Tzika et al. 2015; Shibata et al. 2018). Resources for *I. braminus* have expanded in several steps. A draft genome was generated from Illumina sequencing of genomic DNA (Khedkar et al. 2022). A PacBio full‐length transcriptome later added transcript and isoform information (Zhu et al. 2024). Chromosome and subgenome analyses were then combined with expression and co‐expression across seven tissues in a broader investigation of questions related to parthenogenesis (Lv et al. 2025). Of these advances, the seven‐tissue expression analysis is closest to our study. Both studies compare expression among multiple tissues, but their analytical questions differ. In the earlier study, tissue expression was considered together with chromosome, subgenome, and reproductive questions (Lv et al. 2025). We use a PacBio‐derived transcript reference and a separate Illumina RNA‐seq dataset for tissue‐level quantification. This combination provides a complementary transcriptomic resource for candidate prioritization and hypothesis generation.

Here, we focus on tissue‐level expression and candidate prioritization. Ovary, heart, liver, and skin were sampled from the same three individuals. This allowed tissue differences to be tested while retaining individual identity. From these comparisons, we defined operational candidate tissue‐enriched sets and examined annotation over‐representation. We also asked whether exploratory co‐expression modules remained stable under prespecified sensitivity analyses. Eye reduction is anatomically variable among fossorial squamates (Caprette et al. 2004; Foureaux et al. 2010). Molecular studies also indicate that visual‐system change has not followed a single route across snakes (Davies et al. 2009; Simões et al. 2015, 2016; Schott et al. 2018; Gower et al. 2021; Tang et al. 2021; Peng et al. 2023). The eye‐spot libraries had a different sampling structure from the four‐tissue dataset. Because the three pools overlapped, they remained outside formal tissue inference and were used only to build a descriptive library‐level catalog. The tissue‐resolved candidate sets, descriptive eye‐spot profiles, and stability‐screened co‐expression modules can be used to select targets for functional validation and to design future comparative studies of parthenogenesis, triploid maintenance, fossorial specialization, and visual reduction in reptiles.

## 2 Materials and Methods

### 2.1 Animal Collection and Tissue Sampling

Five adult *Indotyphlops braminus* were collected in the wild in Wangmo County, Guizhou Province, China (25.29° N, 106.31° E), during July–August 2023. Two animals contributed to the PacBio reference‐transcriptome component. The other three were used for Illumina expression profiling. Each animal was housed separately for approximately 1 week. Ventilated plastic containers held loose, slightly moist sand, humus‐rich soil, and leaf litter. A cleaned mineral‐water bottle cap was used as a water dish. Animals were not fed. Conditions were 24°C–28°C, 75%–85% relative humidity, and a natural light cycle.

Sodium pentobarbital was administered at 100 mg kg −1 to induce irreversible deep anesthesia. Terminal tissue collection began only after the absence of a response to an appropriate stimulus had been confirmed. Death was ensured by exsanguination and removal of vital organs. Tissue samples for Illumina sequencing were placed in liquid nitrogen immediately after collection. Ovarian developmental stage was not determined from external appearance. Animal handling and experimental procedures were approved by the Ethics Committee of Guizhou Normal University (authorization number: 20230300015).

### 2.2 Sampling Design for PacBio and Illumina Transcriptome Sequencing

The PacBio component used brain, heart, liver, skin, and muscle material from two animals. Ovary and eye‐spot material were not included. The archived records do not resolve the pooling stage or the final number of PacBio libraries, so neither is specified.

The Illumina dataset comprised 15 paired‐end libraries derived from three animals. Ovary, heart, liver, and skin were sampled separately from each animal, yielding 12 unpooled libraries, with three libraries per tissue. In the analysis files, G, H, L, and S denote ovary, heart, liver, and skin, respectively. G1, H1, L1, and S1 came from individual 1; suffixes 2 and 3 identify the corresponding samples from individuals 2 and 3. This design allowed tissue differences to be analyzed while retaining individual identity.

Bilateral eye‐spot material obtainable from one animal was insufficient for an individual RNA‐seq library, so each of the three eye‐spot libraries (E1–E3) combined bilateral material from two or three of the Illumina animals. Because animals contributed to more than one pool, donor membership overlapped among E1–E3. Dissection was performed under magnification, but a small amount of surrounding tissue could not be excluded. E1–E3 were therefore neither independent biological replicates nor simple technical replicates, and they were not used for formal individual‐level inference (Table S1).

### 2.3 Reference Transcript Processing and Annotation

PacBio data were used to construct the reference transcript set (Rhoads and Au 2015; Wenger et al. 2019). Illumina reads were used only for expression quantification. The archived provider report records circular consensus sequence (CCS) generation with ‐‐min‐passes 1, ‐‐top‐passes 100, ‐‐min‐length 200, ‐‐max‐length 100,000, and ‐‐min‐rq 0.8, followed by CCS filtering with ‐‐rq 0.90, ‐‐np 1, and ‐‐length 200. Primer removal, refinement, and transcript clustering were subsequently performed with lima, isoseq3 refine, and isoseq3 cluster, respectively.

The resulting PacBio consensus transcripts were then clustered with CD‐HIT‐EST 4.8.1 (‐c 0.98 ‐G 0 ‐aL 0.5 ‐aS 0.5 ‐M 0 ‐T 16 ‐d 0) (Fu et al. 2012). Transcripts in the same CD‐HIT‐EST cluster were assigned to a single operational gene unit. These units were not genome‐coordinate‐validated gene loci.

GenomeThreader 1.7.3 used animal protein sequences from Swiss‐Prot as external evidence for coding‐sequence annotation (Gremme et al. 2005). The recorded options were ‐startcodon ‐finalstopcodon ‐gcmincoverage 70 ‐prseedlength 7 ‐prhdist 4 ‐gff3out ‐skipalignmentout. Pfam evidence was not used in this step. Sequences without a GenomeThreader annotation were then processed with TransDecoder 5.5.0 using Pfam 35.0 and Swiss‐Prot evidence (Haas et al. 2013; Mistry et al. 2021; The UniProt Consortium 2025). Results from the two routes were combined. According to the provider report, the longest transcript was selected as the structural‐annotation representative based on the CD‐HIT‐EST cluster mapping. Structural annotations were retained for 41,450 of the 53,489 operational gene units. Predicted protein sequences from these 41,450 representative records were functionally annotated against Swiss‐Prot and the NCBI non‐redundant protein sequence database (NR) with BLASTp 2.10.1+ (Altschul et al. 1997) using an E‐value threshold of 1 × 10–10. For each database, the best hit was retained when sequence identity, query coverage, and subject coverage were each at least 30%. Kyoto Encyclopedia of Genes and Genomes (KEGG) orthology was assigned with Kofam 1.3.0 (Aramaki et al. 2020), and InterProScan 5.22 (Jones et al. 2014) supplied InterPro and Gene Ontology (GO) annotations. The available provider report documented the workflow, software versions listed above, and filtering thresholds; it did not record versions for some Iso‐Seq components or the releases and retrieval dates of the historical Swiss‐Prot and NR databases.

### 2.4 Illumina Preprocessing and Quantification

Paired‐end Illumina reads were processed with fastp 1.3.6 using ‐‐detect_adapter_for_pe, ‐‐trim_front1 5, ‐‐trim_tail1 5, ‐‐trim_front2 5, ‐‐trim_tail2 5, ‐‐cut_right, ‐‐cut_window_size 4, ‐‐cut_mean_quality 20, ‐‐length_required 50, ‐‐n_base_limit 3, ‐‐qualified_quality_phred 20, ‐‐unqualified_percent_limit 10, and ‐‐thread 4 (Chen et al. 2018). Read correction was not enabled. Salmon 1.8.0 quantified the reads against a transcript‐only index built from Rbra.transcripts.fasta with k = 31, libType = A, ‐‐validateMappings, and four threads (Patro et al. 2017); sequence‐, GC‐, and position‐bias options were not enabled, and the index contained no decoy targets. The reference contained 233,716 transcript sequences. The transcript‐to‐gene table covered 233,714 of them. We used tximport 1.30.0 with type = “salmon” and countsFromAbundance = “scaledTPM” to aggregate the estimates to 53,489 operational gene units (Soneson et al. 2015). The output contained gene‐level estimated counts and transcripts per million (TPM) values, not raw integer counts. The separate 41,450‐entry representative‐transcript annotation layer did not define the size of the Salmon reference (Table S2). Per‐library raw and clean pairs, retention percentages, mapping rates, source‐unit identities, and analysis roles are reported in Table S4.

### 2.5 Multidimensional Scaling of Expression Profiles

The multidimensional scaling analysis included the 12 unpooled ovary, heart, liver, and skin libraries. The three pooled eye‐spot libraries were not included because donor animals overlapped among pools. After filtering and trimmed mean of M‐values (TMM) normalization, we generated a multidimensional scaling (MDS) plot with edgeR. The plot used leading log‐fold‐change distances from the 500 common genes selected by plotMDS (gene. selection = “common”) and displayed dimensions 1 and 2. It was used to examine global sample structure and was not treated as a separate hypothesis test (Robinson et al. 2010; Chen et al. 2025).

### 2.6 Differential Expression Among Ovary, Heart, Liver, and Skin

Formal differential‐expression analysis used 12 separate, unpooled libraries: one ovary, one heart, one liver, and one skin library from each of the same three individuals. The three overlapping eye‐spot pools (E1–E3) were excluded from the formal differential‐expression model. The edgeR input used the abundance‐scaled gene‐level estimated counts described above, without a transcript‐length offset. Filtering was performed across the 12 four‐tissue libraries with filterByExpr(group = tissue, min.count = 10, min.total.count = 15, large.*n* = 10, min.prop = 0.7). This retained 30,224 of 53,489 genes. We then applied TMM normalization (Robinson and Oshlack 2010). The edgeR 4.0.2 model was fitted in R 4.3.3 with the design ~ individual + tissue (Robinson et al. 2010; R Core Team 2024). This design kept the correspondence of the same three individuals across the four tissues. Dispersion estimation and quasi‐likelihood fitting used the robust options in estimateDisp and glmQLFit, followed by glmQLFTest (Chen et al. 2016, 2025). We first tested the three tissue coefficients together in a three‐degree‐of‐freedom omnibus test. Six pairwise contrasts were also fixed before testing: H − G, L − G, S − G, L − H, S − H, and S − L. The omnibus test and the six contrasts were treated as seven separate Benjamini–Hochberg (BH) families across the same 30,224 genes (Benjamini and Hochberg 1995). We used a false discovery rate (FDR) threshold of 0.05 and did not add an absolute log2 fold‐change threshold.

### 2.7 Candidate Tissue‐Enriched Sets and Eye‐Spot Profiles

We used the six prespecified pairwise contrasts among the four tissues to define candidate tissue‐enriched gene sets. A gene had to show higher expression in one target tissue than in each of the other three tissues. Each of these three contrasts also had to meet FDR ≤ 0.05 in its own BH family. We did not add an absolute log‐fold‐change threshold. This is the intersection of three existing tests, not a new joint test.

E1–E3 were analyzed only as pooled, library‐level eye‐spot profiles. Their source individuals overlap, so the three libraries were not treated as independent replicates. We ranked the existing tximport TPM values within each library across all 53,489 gene IDs. No detection threshold was used. Pairwise Spearman correlation and cumulative top‐k overlap were used to describe rank similarity. The top‐100 comparison was a descriptive summary, not an expression or significance threshold. We did not average the three libraries or perform hypothesis tests, differential‐expression analysis, enrichment analysis, or WGCNA for the eye‐spot profiles.

### 2.8 GO and KEGG Over‐Representation Analyses

The 30,224 genes tested by edgeR formed the starting universe for Gene Ontology (GO) and Kyoto Encyclopedia of Genes and Genomes (KEGG) over‐representation analyses (ORA). The background for each annotation collection included only genes with an annotation in that collection. For each pairwise contrast, FDR‐significant genes were divided according to the sign of the log2 fold change (logFC). This produced 12 directional foregrounds. We did not use the omnibus result for ORA and did not add a fold‐change threshold. Functional links were assigned through the fixed gene‐to‐Swiss‐Prot accession mapping used in this study (The UniProt Consortium 2025). This mapping does not provide direct annotation for this species and does not establish one‐to‐one orthology.

GO annotations came from UniProtKB release 2026_03, and the GO structure was dated 27 July 2023 (Ashburner et al. 2000; The Gene Ontology Consortium 2023). The annotation mapping contained 237 GO IDs absent from this frozen structure, representing 798 direct gene–GO associations. These IDs were excluded without guessed replacement, and the complete list is provided in Data S18. Biological process (BP), molecular function (MF), and cellular component (CC) terms were tested separately with the classic one‐sided Fisher test in topGO 2.54.0 (nodeSize = 1) (Alexa et al. 2006). Their gene‐level backgrounds contained 14,749, 13,943, and 15,866 genes, respectively. KEGG mapping used release 119.0 and a REST snapshot from 3 September 2026 (Kanehisa and Goto 2000; Kanehisa et al. 2025). We tested 373 reference pathways by a one‐sided hypergeometric test with a background of 6872 genes.

Each direction and annotation collection formed its own BH family. There were 48 gene‐level families, and all terms in a family were adjusted before FDR ≤ 0.05 was applied. Of the 30,224 tested genes, 16,567 had a Swiss‐Prot accession and collapsed to 11,821 unique reviewed accessions for the sensitivity analysis, which retained the same 48‐family design. The accession‐level BP, MF, CC, and KEGG backgrounds contained 10,560, 9933, 11,358, and 5148 accessions, respectively. Across the six contrasts, 170 contrast–accession conflict events were recorded when genes linked to the same accession had opposite signs; the affected accession was removed from both directional foregrounds for that contrast. This analysis did not replace the gene‐level analysis. GO redundancy reduction was used only for display.

### 2.9 Exploratory Co‐Expression Analysis

Weighted gene co‐expression network analysis (WGCNA) was used only as an exploratory analysis (Langfelder and Horvath 2008). It included the 12 unpooled four‐tissue libraries and did not include E1–E3. We calculated TMM‐adjusted log2‐transformed counts per million (log2‐CPM) values with a prior count of 1 for the 30,224 genes retained by the four‐tissue filtering step. Additive individual effects were removed with limma 3.58.1 using removeBatchEffect, with individual ID supplied as batch and a no‐intercept tissue design (model. matrix(~0 + tissue)) supplied as design (Ritchie et al. 2015). The 5000 genes with the highest median absolute deviation were used for network construction; differential‐expression significance was not used for this selection. A candidate central power between 2 and 19 was required to have signed scale‐free fit *R* ^2^ ≥ 0.80, a negative slope, and median connectivity ≥ 5. The central power and its two adjacent powers also each had to have signed *R* ^2^ ≥ 0.75, a negative slope, and median connectivity ≥ 2. No candidate among powers 1–20 met this prespecified rule. We therefore used the backup power of 18 recommended in the WGCNA frequently asked questions for signed networks with fewer than 20 samples and fixed powers 16–20 as the sensitivity range. This choice was not treated as evidence of a successful scale‐free fit. The central network was constructed with WGCNA 1.73 in R 4.3.3 using biweight midcorrelation, signed adjacency, a signed topological overlap matrix, deepSplit = 2, minModuleSize = 30, and mergeCutHeight = 0.25. Additional central settings were maxPOutliers = 0.10, pearsonFallback = “individual,” TOMDenom = “min,” maxBlockSize = 6000, detectCutHeight = 0.995, pamRespectsDendro = TRUE, reassignThreshold = 0, and numericLabels = TRUE. The analysis used seed 20,260,905 and four threads, and the gray category was treated as unassigned (Langfelder and Horvath 2012; R Core Team 2024).

Network stability was checked with nine fixed alternatives. These included powers 16, 17, 19, and 20, deepSplit = 1, mergeCutHeight = 0.20, and three networks that each left out one individual. For each leave‐one‐individual‐out network, the same 5000 genes were retained, and individual‐effect removal was recomputed on the remaining eight libraries using the corresponding individual factor and no‐intercept tissue design. A module was retained only if it contained at least 30 genes and reached a best non‐gray Jaccard overlap of at least 0.50 in every alternative network. Candidate intramodular hub genes were selected only from these stable modules. Each candidate required an absolute module membership of at least 0.70 and a top‐5% rank in the central network, the same mapped module identity in all nine alternatives, a top‐20% rank in every alternative, and a median alternative rank within the top 10%. Module eigengene patterns were summarized by tissue without hypothesis testing. No module *p* values or FDR values were calculated.

## 3 Results

### 3.1 Reference Resource and Expression Matrix

Salmon quantification completed for all 15 Illumina libraries without reported quantification errors. The PacBio‐derived reference contained 233,716 transcript sequences, and 233,714 of them were represented in the transcript‐to‐gene table. Aggregation produced expression estimates for 53,489 operational gene units. Of these 53,489 operational gene units, 41,450 had retained structural annotations and corresponding representative records; this annotation layer was distinct from the Salmon reference. Among these 41,450 representatives, 18,223 (43.96%) matched Swiss‐Prot, 22,745 (54.87%) matched NR, 14,500 (34.98%) received KEGG orthology assignments, 25,885 (62.45%) had InterPro annotations, and 17,700 (42.70%) had GO annotations. In total, 26,791 representatives (64.63%) received at least one functional annotation. The study design and resource hierarchy are summarized in Figure 2. Mapping rates ranged from 80.15% to 90.99% across the 12 libraries representing ovary, heart, liver, and skin (three libraries per tissue; Table S4). The three eye‐spot libraries had lower and more variable mapping rates and are reported separately below (Figure S1).

**FIGURE 2:**
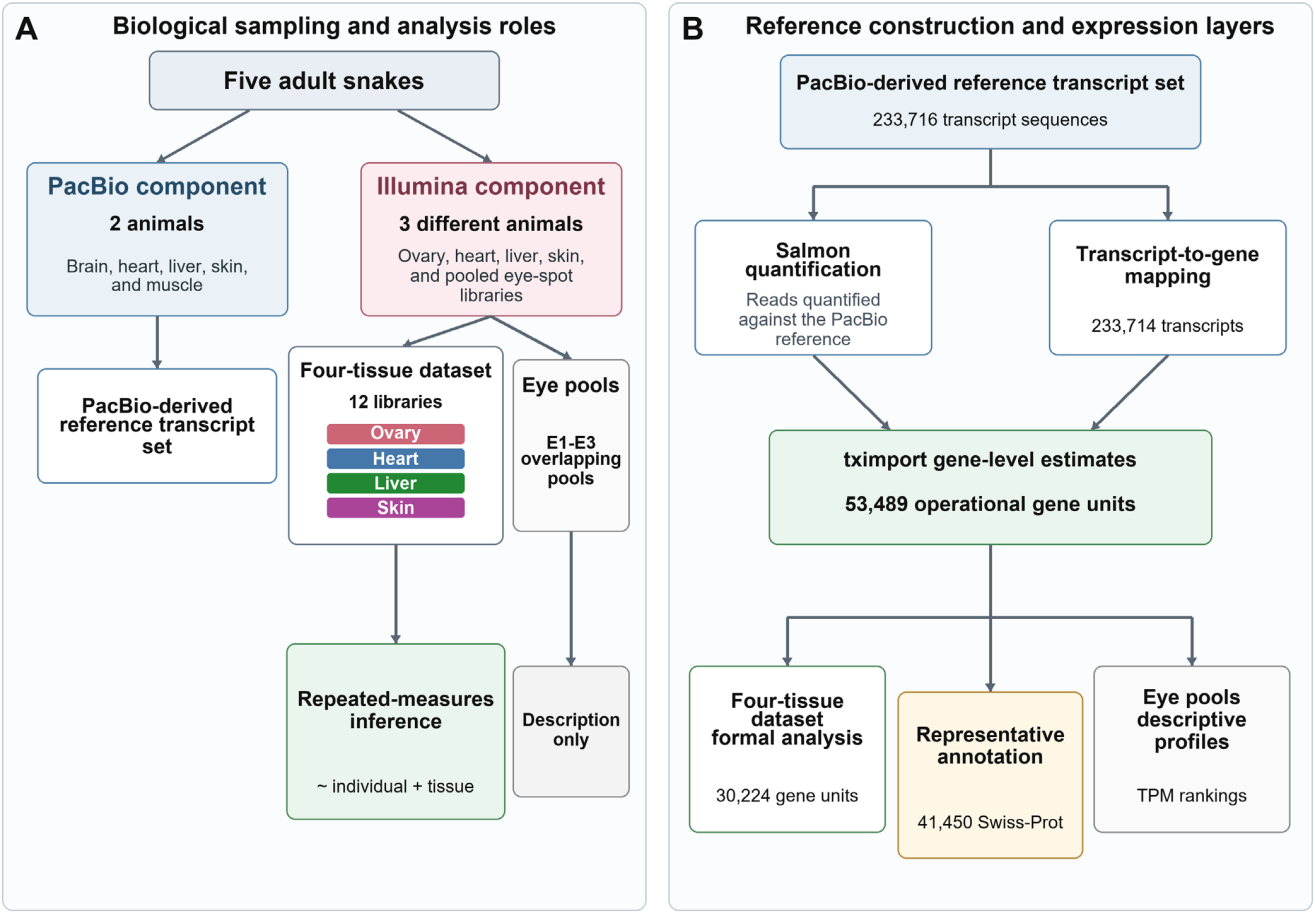
Study design and transcriptomic workflow. (A) Five adult snakes contributed to two distinct sequencing components. Two animals provided brain, heart, liver, skin, and muscle material for PacBio reference‐transcript generation; ovary and eye‐spot material were not included. Three different animals provided the Illumina libraries. Ovary, heart, liver, and skin from these animals formed the 12‐library four‐tissue repeated‐measures set used for formal analysis. E1–E3 were overlapping pooled eye‐spot libraries and were retained for library‐level description only. (B) The PacBio‐derived reference contained 233,716 transcript sequences, and 233,714 transcripts entered the transcript‐to‐gene mapping. Tximport aggregation produced estimates for 53,489 operational gene units. The formal four‐tissue filter retained 30,224 units, whereas the descriptive eye‐pool profiles used the full 53,489‐gene TPM rankings. The representative‐transcript annotation layer contained 41,450 records and was linked through the gene‐to‐Swiss‐Prot accession mapping used in this study. Operational gene units are not genome‐coordinate‐validated loci, and the study did not use a hybrid assembly. TPM, transcripts per million.

### 3.2 Multidimensional Scaling of Expression Profiles Across the Four Tissues

The MDS analysis was restricted to the 12 unpooled libraries used for formal inference. These libraries formed four tissue‐associated groups corresponding to ovary, heart, liver, and skin (Figure 3). H2 and O2 showed more within‐tissue displacement than the other libraries, but both remained in the expected tissue group. All 12 libraries were retained. The pooled eye‐spot libraries were not included in this plot.

**FIGURE 3:**
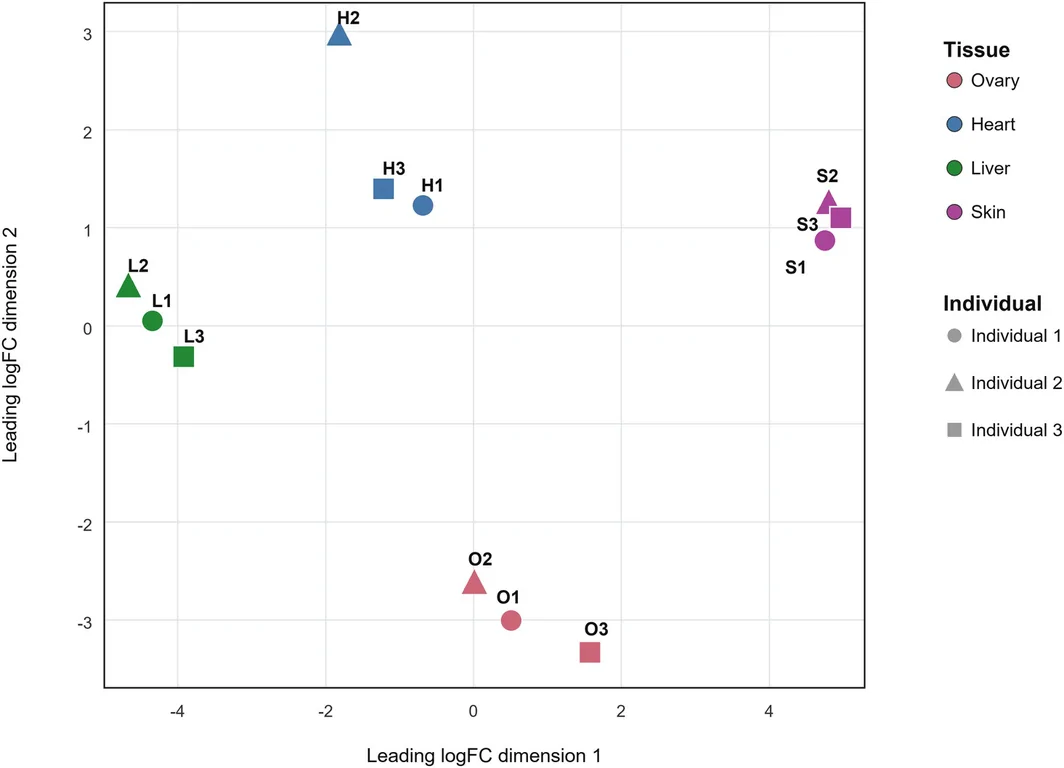
Multidimensional scaling of expression profiles from the 12 formally analyzed tissue libraries. Multidimensional scaling was calculated from the filtered and TMM‐normalized data for the 12 four‐tissue libraries. Colors indicate ovary, heart, liver, and skin, and plotting symbols indicate the three corresponding individuals. Samples grouped primarily by tissue. H2 and O2 showed greater within‐tissue displacement than the other libraries, but both remained within their expected tissue groups and were retained. The pooled eye‐spot libraries were excluded from the formal four‐tissue analysis and are not shown. O1–O3 are display aliases for the source libraries G1–G3. TMM, trimmed mean of M‐values.

### 3.3 Differential Expression Among Ovary, Heart, Liver, and Skin

Formal differential‐expression analysis retained 30,224 of the 53,489 operational gene units. At FDR ≤ 0.05, the three‐degree‐of‐freedom omnibus test identified a tissue effect for 13,849 genes. The six prespecified pairwise contrasts identified 9023 genes for H − G, 11,495 for L − G, 9653 for S − G, 2490 for L − H, 2589 for S − H, and 4950 for S − L (Figure 4; Table 1; Data S1–, S4). Positive and negative logFC counts are shown in the complete summary table. The omnibus test and the six contrasts were corrected as seven separate BH families across the same 30,224 genes. No absolute log‐fold‐change threshold was applied.

**FIGURE 4:**
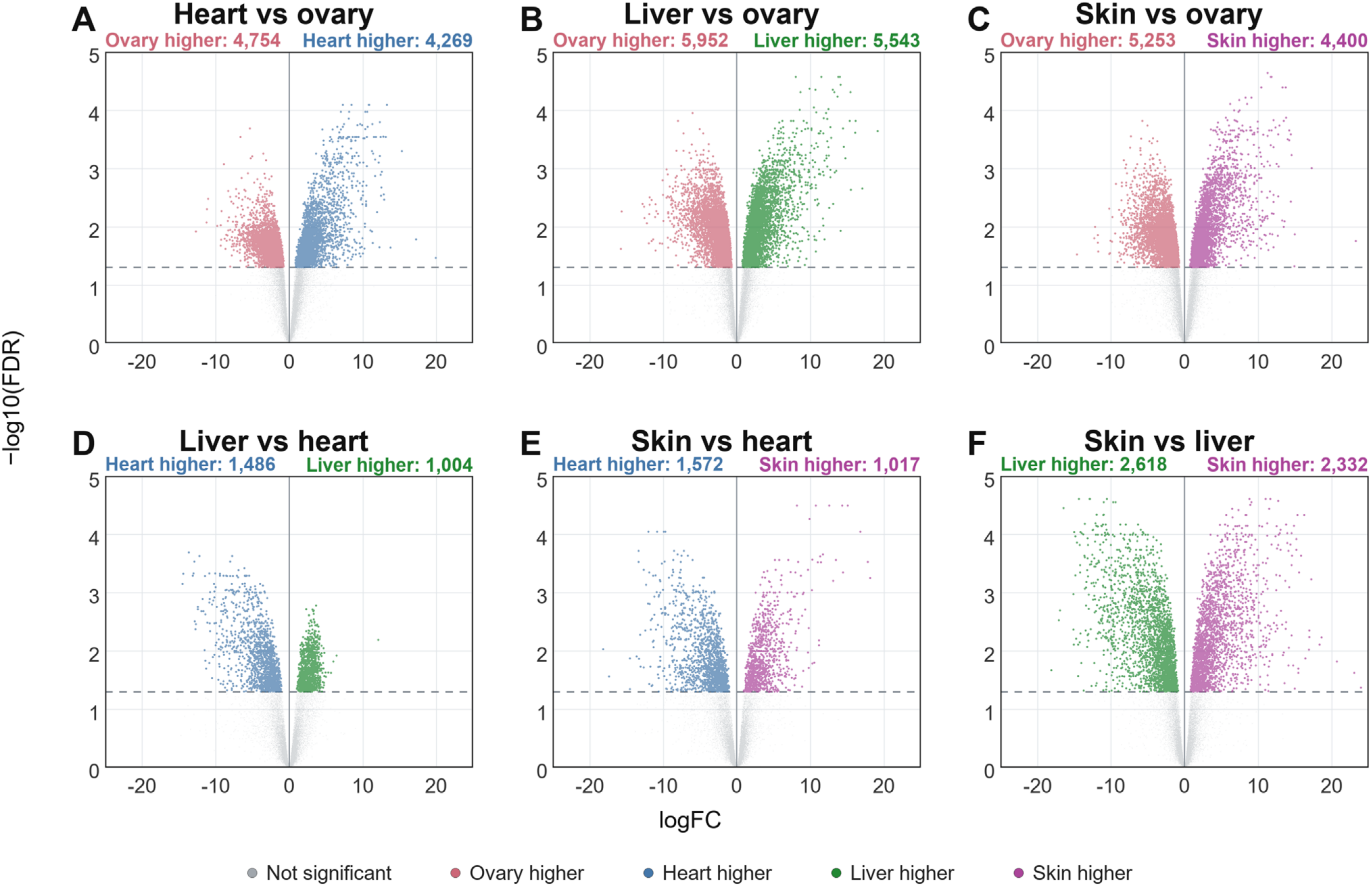
Pairwise differential expression among the four tissues. Volcano plots show the six prespecified pairwise contrasts among ovary, heart, liver, and skin: (A) heart versus ovary, (B) liver versus ovary, (C) skin versus ovary, (D) liver versus heart, (E) skin versus heart, and (F) skin versus liver. Each panel contains the same 30,224 operational gene units. The dashed line marks FDR = 0.05; no absolute logFC cutoff was applied. Point colors indicate the tissue with higher expression, and the labels report the significant counts in each direction. FDR correction was performed separately within each contrast, so FDR values were not pooled across panels. The overlapping eye‐spot libraries were excluded from these formal comparisons. Abbreviations: FDR, false discovery rate; logFC, log2 fold change. Formal differential‐expression inference used the 12 ovary, heart, liver, and skin libraries and 30,224 retained operational gene units with the design ~individual + tissue. One three‐degree‐of‐freedom tissue omnibus test and six prespecified pairwise contrasts were evaluated. Each test was corrected as a separate BH family at FDR ≤ 0.05. No absolute log‐fold‐change threshold was applied. For pairwise contrasts, positive logFC denotes higher expression in the tissue named first. BH, Benjamini–Hochberg; df, degrees of freedom; FDR, false discovery rate; logFC, log2 fold change; NA, not applicable. NA is not zero and is used where direction‐specific counts do not apply to the omnibus test.

**TABLE 1:** Formal differential‐expression summary for the 12 four‐tissue libraries.

Test	df	Genes tested	Significant at FDR ≤ 0.05	Positive direction	Positive genes	Negative direction	Negative genes
Tissue omnibus (3 df)	3	30,224	13,849	NA	NA	NA	NA
Heart versus ovary	1	30,224	9023	Heart > ovary	4269	Ovary > heart	4754
Liver versus ovary	1	30,224	11,495	Liver > ovary	5543	Ovary > liver	5952
Skin versus ovary	1	30,224	9653	Skin > ovary	4400	Ovary > skin	5253
Liver versus heart	1	30,224	2490	Liver > heart	1004	Heart > liver	1486
Skin versus heart	1	30,224	2589	Skin > heart	1017	Heart > skin	1572
Skin versus liver	1	30,224	4950	Skin > liver	2332	Liver > skin	2618

### 3.4 Candidate Tissue‐Enriched Sets and Descriptive Eye‐Spot Profiles

We next intersected the three relevant pairwise contrasts for each tissue in the four‐tissue results. This identified 4228 candidate tissue‐enriched genes: 2521 for ovary, 281 for heart, 770 for liver, and 656 for skin (Figure 5; Data S5–, S6). The four sets did not overlap. These counts summarize an intersection of three existing pairwise tests for each tissue. They are not the result of a new joint test and do not establish tissue‐exclusive expression.

**FIGURE 5:**
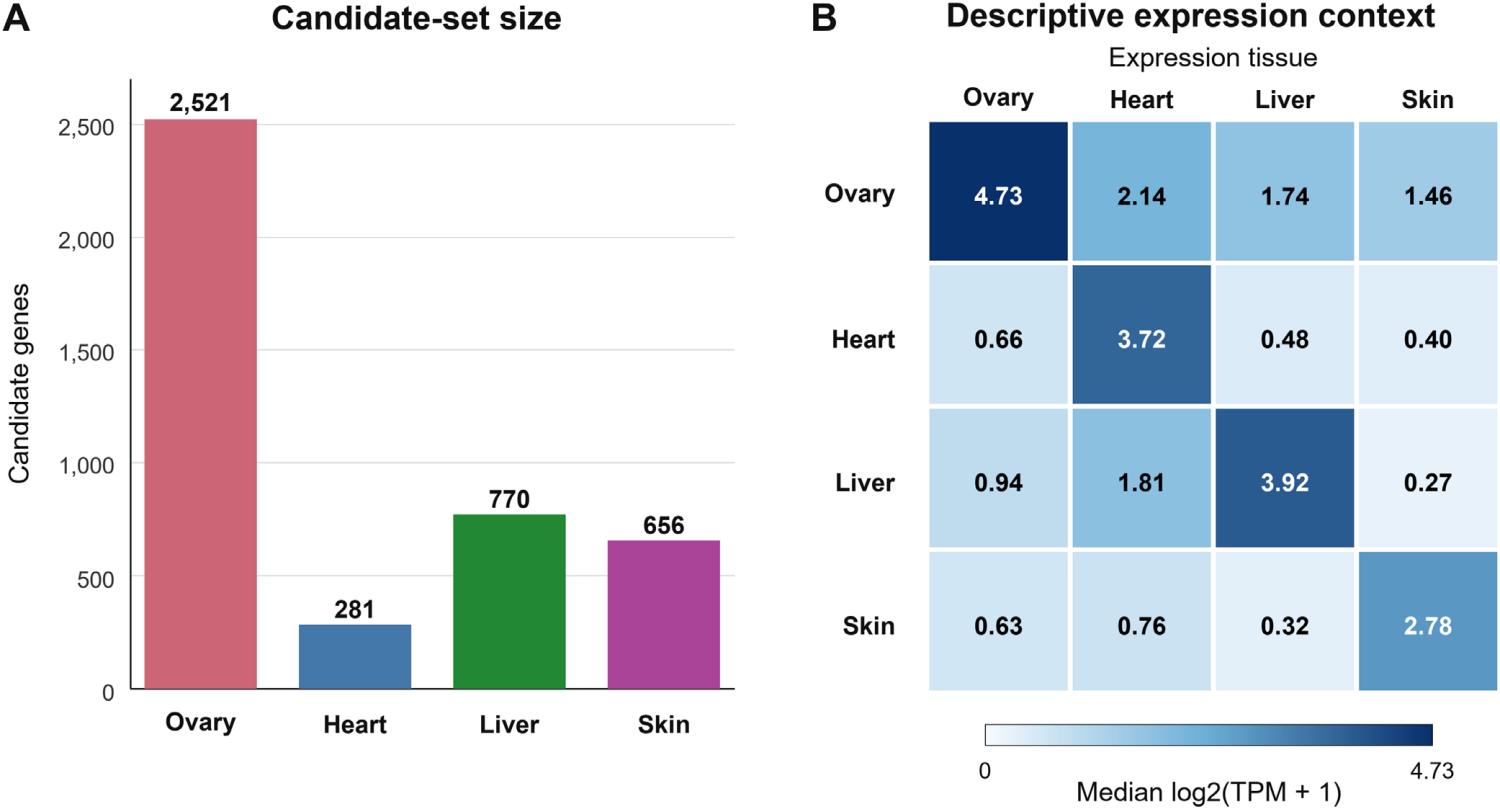
Candidate tissue‐enriched genes and their descriptive expression context. (A) A gene entered a tissue candidate set when it showed higher expression in that tissue in all three relevant prespecified pairwise contrasts and each contrast met FDR ≤ 0.05 within its own BH family. This rule identified 2521 candidates for ovary, 281 for heart, 770 for liver, and 656 for skin. The four sets were non‐overlapping. These counts are intersections of existing pairwise results, not a new joint test, and no absolute log‐fold‐change threshold was applied. (B) Each cell shows the median log2(TPM + 1) across genes assigned to the indicated candidate set and tissue. The TPM summary is descriptive and was not used as an additional significance test. Abbreviations: BH, Benjamini–Hochberg; FDR, false discovery rate; TPM, transcripts per million.

E1, E2, and E3 were overlapping pools and were analyzed only as library‐level eye‐spot profiles. Their Salmon mapping rates were 49.81%, 47.76%, and 26.03%, respectively. Despite the lower mapping rate for E3, the full TPM ranks were similar across the three libraries, with pairwise Spearman correlations from 0.9585 to 0.9795. The three top‐100 lists shared 81 genes and contained 119 genes in their union, giving a Jaccard index of 0.6807 (Figure S1; Table S4; Data S7–, S11). These values support a descriptive candidate catalog. They do not provide *p* values, FDR, evidence of eye‐spot‐specific expression, or evidence of visual function.

### 3.5 Representative ORA Terms Retained in the Sensitivity Analysis

The gene‐level analysis produced 6888 significant term records across 48 prespecified BH families. The corresponding accession‐level sensitivity analysis produced 4162 significant records across a parallel set of 48 families. These totals count term records across separate families and are not effect sizes. Six terms were retained for concise main‐text presentation because they were significant in both the gene‐level analysis and the accession‐level sensitivity analysis. The display choice also considered GO redundancy where applicable, the degree to which multiple gene units collapsed onto the same accession, direct relevance to the sampled tissues and study questions, and the risk of overinterpretation; it was not based on FDR rank and did not alter any ORA result (Table 2).

**TABLE 2:** Representative annotation terms retained for main‐text interpretation.

Term ID	Collection	Comparison	Direction	Term	Gene hits	Gene FDR	Accession hits	Accession FDR
GO:0030280	GO molecular function	Skin versus ovary	Skin > ovary	Structural constituent of skin epidermis	14	2.404907e‐08	7	1.035166e‐02
GO:0014704	GO cellular component	Liver versus heart	Heart > liver	Intercalated disc	15	2.236926e‐07	14	2.659360e‐07
GO:0005782	GO cellular component	Liver versus heart	Liver > heart	Peroxisomal matrix	15	7.903183e‐08	12	4.461200e‐06
map03430	KEGG pathway	Heart versus ovary	Ovary > heart	Mismatch repair	20	2.304837e‐08	16	5.326891e‐06
map03030	KEGG pathway	Liver versus ovary	Ovary > liver	DNA replication	28	1.960575e‐06	24	9.137034e‐05
map04382	KEGG pathway	Skin versus heart	Skin > heart	Cornified envelope formation	27	4.595803e‐09	18	3.485699e‐05

*Note:* Six terms were retained for concise main‐text presentation after the complete prespecified ORA analyses. Gene‐level ORA was the primary analysis and accession‐level ORA was the sensitivity analysis. FDR values were calculated separately within each combination of contrast direction and annotation collection at each annotation level. Hit counts denote members of the corresponding foreground set associated with each term. KEGG entries are reference pathways assigned through the gene‐to‐Swiss‐Prot accession mapping used in this study and do not demonstrate pathway activity, tissue‐exclusive function, or a causal mechanism. For concise display, the six terms were selected from results significant at both levels by considering GO redundancy where applicable, the extent to which gene‐level hits mapped to the same Swiss‐Prot accessions, direct relevance to the sampled tissues and study questions, and the risk of overinterpretation. This display choice was not based on FDR rank and did not alter any ORA result; complete results are in Data S12–, S18. FDR, false discovery rate; GO, Gene Ontology; KEGG, Kyoto Encyclopedia of Genes and Genomes; ORA, over‐representation analysis.

In the ovary‐higher direction of H − G, the KEGG mismatch repair reference pathway included 20 gene hits and 16 accession hits. In the ovary‐higher direction of L − G, the KEGG DNA replication reference pathway included 28 genes and 24 accessions. Genes higher in skin than ovary included the GO term structural constituent of skin epidermis, with 14 genes and 7 accessions. Two terms were retained from the heart–liver comparison: intercalated disc for genes higher in heart than liver (15 genes and 14 accessions) and peroxisomal matrix for genes higher in liver than heart (15 genes and 12 accessions). The KEGG cornified envelope formation reference pathway was retained for genes higher in skin than heart, with 27 genes and 18 accessions. No term from the S − L comparison was selected for main‐text interpretation; the broad KEGG cytoskeleton in muscle cells reference pathway is reported only in Table S3. These results reflect annotation over‐representation based on the gene‐to‐Swiss‐Prot accession mapping used in this study. They do not demonstrate pathway activation, tissue‐exclusive function, or a causal mechanism. Complete ORA outputs and sensitivity records are provided in Data S12–, S18.

### 3.6 Exploratory Co‐Expression Analysis

The prespecified scale‐free topology criterion was not met over powers 1–20. We therefore used a soft‐thresholding power of 18 for the signed exploratory network and did not treat it as evidence of a successful scale‐free fit. The central network contained 11 non‐gray modules. Figure 6 shows the full module assignment and the descriptive eigengene patterns; three modules met the stability rule across all nine fixed alternative networks and contained 1904 genes in total. The other eight modules failed at least one prespecified stability condition and were not retained for interpretation. Fifty‐eight candidate intramodular hub genes also passed the rank and identity criteria in the central and all alternative networks (Data S19–, S24). No individual hub is highlighted in the main text, and no module enrichment analysis was performed.

**FIGURE 6:**
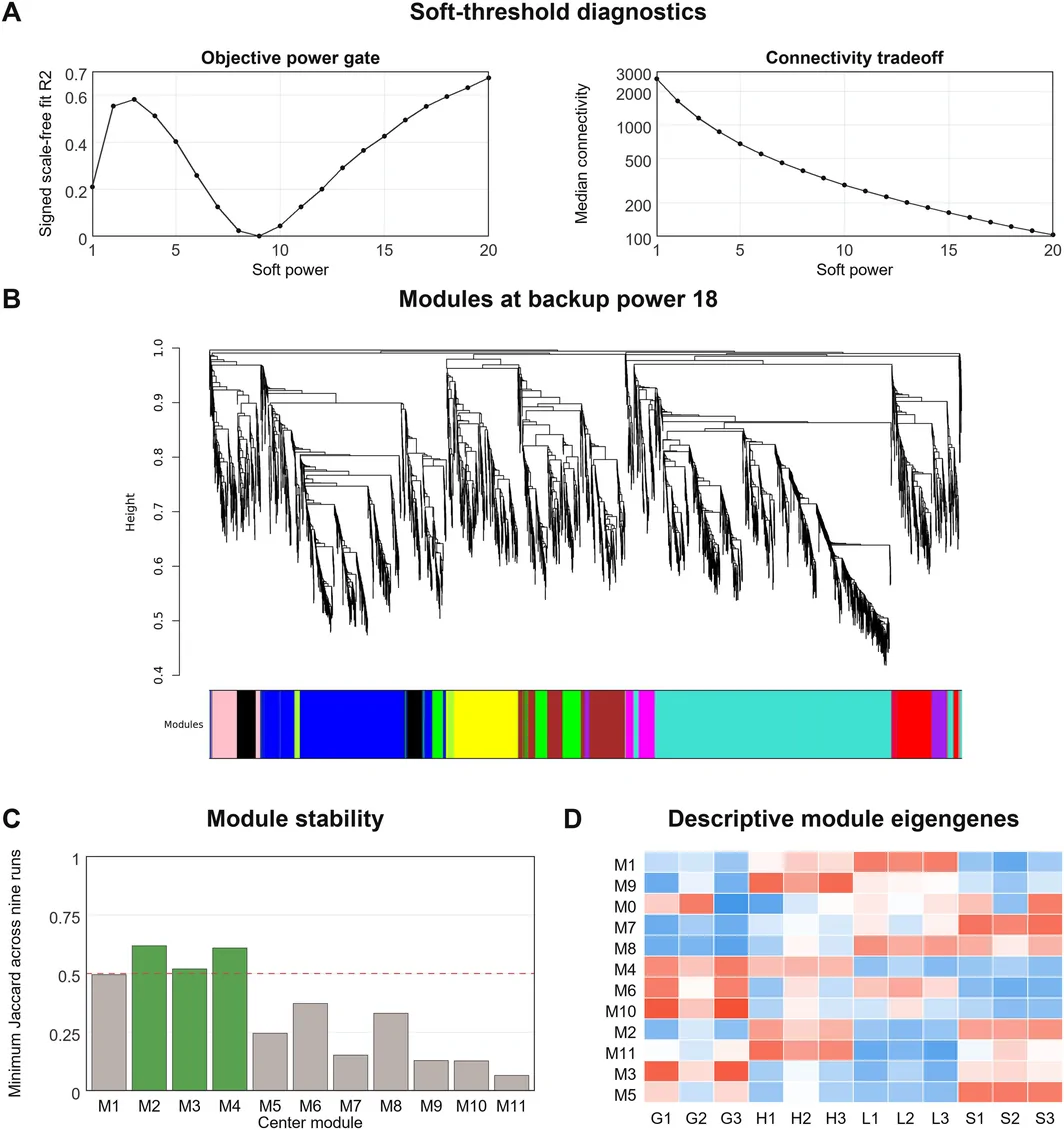
Exploratory signed co‐expression analysis of 12 four‐tissue RNA‐seq libraries. (A) Signed scale‐free fit and median connectivity across candidate soft‐thresholding powers. The prespecified scale‐free topology criterion was not met over powers 1–20. (B) Hierarchical gene clustering and module assignment for the documented small‐sample fallback power of 18. (C) Minimum module‐mapping Jaccard values across nine fixed alternative networks; only M2, M3, and M4 met the threshold of 0.50 in every run. (D) Module eigengenes across the 12 libraries, shown descriptively without module‐tissue P or FDR values. G1–G3 are the source‐library codes for ovary samples, and M0 denotes the gray/unassigned module. In panel D, blue and red denote negative and positive eigengene values, respectively, on a common symmetric color scale centred at zero. No module enrichment analysis or validated regulatory claim was made. FDR, false discovery rate.

## 4 Discussion

Previous work has already provided a draft genome and a PacBio full‐length transcriptome for *I. braminus* (Khedkar et al. 2022; Zhu et al. 2024). A later study combined chromosome and subgenome analyses with expression and co‐expression across seven tissues (Lv et al. 2025). The present study extends these resources through a different sampling and analytical design. We used a PacBio‐derived transcript reference to quantify expression in a separate Illumina RNA‐seq dataset. Ovary, heart, liver, and skin were sampled from the same three individuals. The three eye‐spot libraries were overlapping pools. We therefore used the four‐tissue data for formal repeated‐measures inference and kept the eye‐spot data descriptive.

Tissue identity was associated with broad expression differences among ovary, heart, liver, and skin. Broad tissue‐associated expression structure has also been reported across vertebrate transcriptomes (Kryuchkova‐Mostacci and Robinson‐Rechavi 2017; Cardoso‐Moreira et al. 2019; De Mello et al. 2021). The omnibus test identified a tissue effect for 13,849 of 30,224 tested genes. Across the six pairwise contrasts, significant gene counts ranged from 2490 to 11,495 at FDR ≤ 0.05. The four tissues therefore had broadly different expression profiles after individual identity was retained in the analysis. For candidate prioritization, we applied a narrower operational rule. For each target tissue, expression had to be higher than in all three other tissues. All three contrasts also had to meet FDR ≤ 0.05. This intersection yielded 4228 candidate tissue‐enriched genes. This rule identifies candidate tissue‐enriched genes but does not establish strict tissue‐specific or tissue‐exclusive expression. Candidate numbers differed among tissues, but we do not assign a mechanism to this difference. The resulting sets are used to prioritize genes for later validation.

We next examined annotation over‐representation in the directional sets of differentially expressed genes from the six prespecified pairwise tissue comparisons. Six terms were significant in the primary gene‐level analysis and remained significant after genes sharing a Swiss‐Prot accession were collapsed. Two of the retained terms occurred in ovary‐higher directions: the KEGG DNA replication and mismatch repair reference pathways assigned through the gene‐to‐Swiss‐Prot accession mapping used in this study. Premeiotic endoreplication has been documented in obligately parthenogenetic reptiles, and mismatch repair family proteins have established roles in meiotic crossover formation in model systems (Manhart and Alani 2016; Dedukh et al. 2022, 2024). These links make both terms relevant to reproductive questions, but they do not identify the mechanism used by *I. braminus* . More broadly, cytological studies of parthenogenetic reptiles have identified different routes of meiotic modification or ploidy restoration (Kearney et al. 2009; Lutes et al. 2010; Newton et al. 2016; Fujita et al. 2020; Ryakhovsky et al. 2021). These comparisons provide context, not validation of a mechanism in *I. braminus* . Skin‐higher directions included structural constituent of skin epidermis and cornified envelope formation. These skin‐related candidates provide specific targets for future matched comparisons between fossorial and non‐fossorial lineages, although the present within‐species tissue contrasts do not attribute them to fossorial adaptation. Intercalated disc occurred in a heart‐higher direction, and peroxisomal matrix in a liver‐higher direction. ORA identifies over‐represented annotations; here, the six retained terms were used only to organize and prioritize tissue‐associated candidates and generate hypotheses (Zhao and Rhee 2023). This analysis does not measure pathway activity or establish causal tissue mechanisms.

Eye reduction varies anatomically among fossorial squamates (Caprette et al. 2004; Foureaux et al. 2010). In other blind‐snake lineages, visual reduction is uneven across molecular components. Several cone‐phototransduction genes are absent in *Anilios*, whereas rod‐associated components remain detectable; comparative genomic work has also reported losses and reduced expression among eye‐development and visual‐perception genes in other blind snakes (Gower et al. 2021; Peng et al. 2023). Against this background, the eye‐spot libraries were examined separately at the library level. Their mapping rates differed, particularly for E3. Even so, the full TPM rankings were similar. Pairwise rank correlations ranged from 0.9585 to 0.9795, and 81 genes were shared by all three top‐100 lists. The consistent rankings support a descriptive candidate catalog at the library level. E1–E3 remain pooled libraries, not independent biological replicates. Each library pooled bilateral material from two or three animals, and source individuals were reused across E1–E3. The dissected material may also include tissue surrounding the eye spot. For these reasons, the eye‐spot libraries were not used for differential expression or enrichment. These data therefore do not establish eye‐specific expression or visual function. Linking individual candidates to eye biology will require independent sampling and anatomical validation.

Three of the 11 non‐gray modules remained above the overlap threshold in all nine alternative networks. They contained 1904 genes, including 58 genes that passed the cross‐network rank and identity rules for candidate intramodular hubs. This retained subset came from an exploratory network of 12 libraries from three individuals. The prespecified scale‐free topology criterion was not met over powers 1–20. Power 18 was used as a documented fallback for a signed small‐sample network, not as evidence that the rule had passed. Given the small dataset and the known sample‐size dependence of RNA‐seq co‐expression networks (Mason et al. 2009; Gaiteri et al. 2014; Ballouz et al. 2015), the stability checks restricted follow‐up to a smaller candidate subset. The retained modules and candidate intramodular hubs nevertheless remain exploratory.

The closest comparison is a study of *I. braminus* (Lv et al. 2025), which combined chromosome and subgenome analyses with expression across seven tissues and an ovarian comparison with the sexually reproducing *Argyrophis diardii* . Its genome‐based gene framework, sampling scheme, and reproductive comparison differ from our transcript‐cluster units and individual‐aware contrasts among four tissues within *I. braminus* . These datasets can inform the selection of reproductive candidates, but quantitative comparisons require harmonized gene assignments and sampling metadata. Broader reptile resources address different questions. A garter snake transcriptome was assembled from normalized, sex‐specific RNA pools; venom‐gene homolog expression was examined across diverse Burmese python tissues using a genome‐based framework; and a broad sauropsid sequence resource was developed from normalized libraries spanning adult tissues and embryonic stages (Schwartz et al. 2010; Reyes‐Velasco et al. 2015; Tzika et al. 2015). These studies provide comparative background, but their taxa, pooling, developmental composition, reference frameworks, and analytical aims do not reproduce our four‐tissue design.

Studies of other parthenogenetic reptiles help identify relevant evolutionary questions. Meiosis in parthenogenetic whiptail lizards was shown to begin with twice the number of chromosomes, with bivalents forming between sister chromosomes; ovarian transcriptomes have also been reported for the parthenogenetic lizard *Darevskia unisexualis* and its parental sexual taxa (Lutes et al. 2010; Ryakhovsky et al. 2021). These findings highlight DNA replication, chromosome pairing, and repair‐related candidates for follow‐up, but differences in ancestry, ploidy, and reproductive cytology prevent direct extrapolation to *I. braminus* . All three animals in the formal Illumina comparison were *I. braminus* and shared the same reproductive mode and fossorial lifestyle; neither factor varied within the study, and no external comparator was included. The observed tissue differences therefore cannot be assigned specifically to parthenogenesis, triploidy, or fossoriality. Testing these hypotheses will require independently sampled and histologically staged ovaries, appropriate sexual comparators, explicit orthology assignments (Gabaldón and Koonin 2013), and a common quantification framework. Fossorial or visual hypotheses will additionally require homologous tissues from multiple lineages and targeted molecular, cytological, anatomical, or physiological validation. Within these limits, the expression matrix, candidate sets, annotation results, and stability‐screened modules provide testable starting points.

## 5 Conclusion

This study adds a multi‐tissue expression resource for *I. braminus* by combining a PacBio‐derived transcript reference with a separate Illumina RNA‐seq dataset. Ovary, heart, liver, and skin were sampled from the same three individuals, and individual identity was retained in the tissue contrasts. These contrasts identified 4228 candidate tissue‐enriched genes. The complete differential‐expression outputs and annotation over‐representation results can be used to organize and prioritize candidates, while the descriptive eye‐spot catalog and stability‐screened exploratory modules provide additional targets for follow‐up. Together with existing genomic and transcriptomic resources, these data can support cross‐species comparisons once orthology and sampling conditions are aligned. More precise ovary and eye‐spot sampling, additional independent animals, matched comparators, and molecular, cellular, or functional experiments will be required to test mechanistic hypotheses concerning reproduction and fossorial or visual traits.

## Author Contributions

**Kexin Sun:** conceptualization, methodology, software, formal analysis, investigation, writing – original draft, visualization. **Xiang Ji:** validation, data curation, investigation. **Jing Lu:** investigation, validation. **Anqiong Lu:** investigation, data curation. **Yuqian Wang:** resources, supervision, writing – review and editing. **Fei Zhu:** project administration, funding acquisition, writing – review and editing, supervision.

## Funding

The research design and data collection were financially supported by the Joint Fund of the National Natural Science Foundation of China and the Karst Science Research Center of Guizhou Province (grant number U1812401) and the new seedling plans of Guizhou Normal University ([2021]B03).

## Ethics Statement

All samples were collected following Chinese regulations for the Implementation of the Protection of Terrestrial Wild Animals (State Council Decree (1992) No. 13). All the experiments and methods were performed in accordance with the Animal Research: Reporting of In Vivo Experiments (ARRIVE) guidelines (Percie Du Sert et al. 2020). All the methods were carried out in accordance with relevant guidelines and regulations. Animal handling and experimental procedures were approved by the Ethics Committee of Guizhou Normal University (Permission number: 20230300015).

## Conflicts of Interest

The authors declare no conflicts of interest.

## Supporting information


**Data S1:** Complete 3‐df tissue omnibus results.

**Data S2:** Significant 3‐df tissue omnibus results.

**Data S3:** Complete results for the six prespecified pairwise contrasts.

**Data S4:** Significant results for the six prespecified pairwise contrasts.

**Data S5:** Counts for the four candidate tissue‐enriched sets.

**Data S6:** Complete candidate‐set membership with contrast‐level evidence.

**Data S7:** Mapping rates for the pooled eye‐spot libraries E1–E3.

**Data S8:** Full TPM rankings for E1–E3.

**Data S9:** Full‐rank Spearman correlations among E1–E3.

**Data S10:** Cumulative top‐k overlap across all k for E1–E3.

**Data S11:** Per‐gene eye‐pool rank robustness and four‐tissue expression context.

**Data S12:** Complete gene‐level ORA results across the 48 direction‐by‐collection families.

**Data S13:** Significant gene‐level ORA results.

**Data S14:** Complete accession‐collapsed sensitivity ORA results across the 48 families.

**Data S15:** Significant accession‐collapsed sensitivity ORA results.

**Data S16:** Family‐level counts for the primary and sensitivity ORA analyses.

**Data S17:** Retained, lost, and new term statuses in the accession‐collapsed sensitivity analysis.

**Data S18:** GO identifiers absent from the frozen GO structure and excluded from ORA.

**Data S19:** Power‐18 WGCNA module assignments and kME values.

**Data S20:** Central WGCNA network module sizes.

**Data S21:** Jaccard‐based module mappings across nine alternative WGCNA networks.

**Data S22:** Minimum and median Jaccard stability summaries.

**Data S23:** The complete set of 58 candidate intramodular hubs.

**Data S24:** WGCNA module eigengenes by sample.

**Figure S1:** Descriptive summaries of the three pooled eye‐spot libraries, including Salmon mapping rates, full‐rank Spearman correlations, cumulative top‐k rank overlap, and comparisons with the four‐tissue expression ranks.

**Table S1:** Sample and library design.

**Table S2:** Reference transcript and annotation hierarchy.

**Table S3:** Supplement‐only representative annotation terms.

**Table S4:** Per‐library Illumina processing and analysis roles.

## Data Availability

The Illumina RNA‐seq reads have been deposited in the NCBI Sequence Read Archive under BioProject PRJNA1416658 (https://www.ncbi.nlm.nih.gov/bioproject/1416658) and SRA Study accession SRP671497. All 15 runs are publicly available. The PacBio Iso‐Seq reads used to generate the reference transcript set are publicly available through NCBI BioProject PRJNA951905 (https://www.ncbi.nlm.nih.gov/bioproject/951905), with SRA Run accession SRR24061511. Processed data supporting the results accompany this article as Supporting Information S1 and Data S1–, S24.
